# Dissecting Tissue Compartment-Specific Protein Signatures in Primary and Metastatic Oropharyngeal Squamous Cell Carcinomas

**DOI:** 10.3389/fimmu.2022.895513

**Published:** 2022-05-16

**Authors:** Habib Sadeghirad, James Monkman, Ahmed M. Mehdi, Rahul Ladwa, Ken O’Byrne, Brett G. M. Hughes, Arutha Kulasinghe

**Affiliations:** ^1^ The University of Queensland Diamantina Institute, The University of Queensland, Woolloongabba, QLD, Australia; ^2^ Queensland Cyber Infrastructure Foundation Ltd., QCIF Facility for Advanced Bioinformatics, Brisbane, QLD, Australia; ^3^ Princess Alexandra Hospital, Woolloongabba, QLD, Australia; ^4^ Faculty of Medicine, University of Queensland, Herston, QLD, Australia; ^5^ Cancer Care Services, Royal Brisbane and Women’s Hospital, Herston, QLD, Australia

**Keywords:** oropharangeal cancer, spatial proteomics, head and neck cancer, metastasis, lymph node metastasis, digital spatial profiling

## Abstract

Head and neck squamous cell carcinoma (HNSCC) often presents with locoregional or distant disease, despite multimodal therapeutic approaches, which include surgical resection, chemoradiotherapy, and more recently, immunotherapy for metastatic or recurrent HNSCC. Therapies often target the primary and nodal regional HNSCC sites, and their efficacy at controlling occult distant sites remains poor. While our understanding of the tumor microenvironment conducive to effective therapies is increasing, the biology underpinning locoregional sites remains unclear. Here, we applied targeted spatial proteomic approaches to primary and lymph node metastasis from an oropharyngeal SCC (OPSCC) cohort to understand the expression of proteins within tumors, and stromal compartments of the respective sites in samples of both matched and unmatched patients. In unmatched analyses of n = 43 primary and 11 nodal metastases, our data indicated that tumor cells in nodal metastases had higher levels of Ki-67, PARP, BAD, and cleaved caspase 9, suggesting a role for increased proliferation, DNA repair, and apoptosis within these metastatic cells. Conversely, in matched analyses (n = 7), pro-apoptotic markers BIM and BAD were enriched in the stroma of primary tumors. Univariate, overall survival (OS) analysis indicated CD25 in tumor regions of primary tumors to be associated with reduced survival (HR = 3.3, p = 0.003), while progesterone receptor (PR) was associated with an improved OS (HR = 0.33, p = 0.015). This study highlights the utility of spatial proteomics for delineating the tumor and stromal compartment composition, and utility toward understanding these properties in locoregional metastasis. These findings indicate unique biological properties of lymph node metastases that may elucidate further understanding of distant metastatic in OPSCC.

## Introduction

Head and neck squamous cell carcinoma (HNSCC) is the 7th leading cause of cancer worldwide, with approximately 890,000 new cases and 450,000 deaths (1). HNSCC is considered a heterogeneous malignancy arising from the upper aerodigestive tract, particularly from the squamous mucosal line. The lip, oral and nasal cavity, paranasal sinuses, larynx, nasopharynx, oropharynx, and hypopharynx are the areas involved in HNSCC (1). Several risk factors contribute to the development of HNSCC, including both tobacco and alcohol consumption. Viral-driven HNSCC is also found, with Epstein–Barr Virus (EBV) and human papillomavirus (HPV) responsible for the nasopharynx and oropharynx malignancies, respectively (1).

Oropharyngeal SCC (OPSCC) is responsible for a quarter of HNSCC (2), with a tendency to occur more commonly in non-smokers and frequent nodal involvement. Treatment of OPSCC is aimed at curing and organ preservation using a multimodality approach. In locoregional disease, patients are often treated with chemoradiation. Most notably, HPV-induced OPSCC has a better prognosis compared to HPV-negative OPSCC, namely, better radiation sensitivity and overall survival. The 5-year survival rate for patients with HPV-negative and HPV-positive OPSCC is 46 and 57.4%, respectively (3).

In approximately two-thirds of OPSCC patients, locoregional metastasis has been reported. Advanced nodal status, especially extranodal extension, is a poor prognostic predictor, and defining the molecular phenotypes of these multiple sites is important to understand not only the primary tumor, but also the influence of lymph node metastasis to develop effective therapies (4). To gain a deeper understanding of the primary and metastatic tissues, studies of the tumor microenvironment (TME) are needed, ideally by comparing primary and lymph node metastasis, and where possible, where these are from matched patients. The identification and characterization of potential TME biomarkers could have significant predictive and prognostic value in the treatment selection for OPSCC patients (5). However, this can be challenging due to inter- or intra-tumoral heterogeneity (6). The cellular composition and molecular interactions between the TME and the host immune cells could play a role in disease progression and treatment resistance. It is thought that locoregional metastasis are immune-cold and therefore treatment-resistant to any immuno-modulatory treatment strategies (1, 7).

To delineate primary and nodal disease in OPSCC, we applied digital spatial profiling (DSP) of the TME using targeted-panel multiplex immunohistochemistry of tumor- and stromal-compartments. Our study found in unmatched analyses that the tumor compartments of the primary were enriched for ARG1, PD-L1, and nodal metastases were enriched for Ki-67, PARP, BAD, and cleaved caspase 9. Stromal compartments of the primary were enriched for VISTA and IDO1. In matched analyses, BIM and BAD were enriched in the stroma of primary tumors. Furthermore, protein signatures were identified to discriminate matched primary/nodal tissues, and survival associations were investigated compartmentally by tumor sample site.

## Material and Methods

This study has the approval of the Queensland University of Technology Human Research Ethics Committee (UHREC #2000000494) and University of Queensland ratification. A tissue microarray (TMA) of OPSCC specimens was sourced from the Tristar Technologies Group (USA) and contained forty-three primary tumors, eleven nodal metastases, and seven matched pairs of primary tumor/nodal metastases with concordant clinicopathological annotations.

### Nanostring GeoMx Digital Spatial Profiling (DSP)

The TMA slides from OPSCC samples were obtained and analyzed using the Nanostring GeoMX Digital Spatial Profiling (DSP) technology by the Systems Biology and Data Science Group at Griffith University (Gold Coast, Australia). Pan-cytokeratin and CD45 were the visualization markers used by the instrument to stain the tumor and lymphocytes, respectively. A protein panel of 68 antibodies was used, namely, immune activation, pan-tumor, immuno-oncology (IO) drug target, cell death, immune cell typing, human immune cell core panel, and PI3K/AKT panels. The slides were prepared according to the instructions of the manufacturer, and the tumor/stroma distinction was achieved by masking on PanCK^+^ or PanCK^−^ regions, respectively. Using the Nanostring nCounter^®^ platform, antibody barcodes were counted in accordance with the instructions of the manufacturer. In DSP analysis, external RNA Controls Consortium (ERCC) QC was employed to prepare the data for further bioinformatic analysis.

### Bioinformatic Analysis

Data analysis was conducted in collaboration with the Queensland Cyber Infrastructure Foundation (QCIF, QLD, Australia). The quality of data was investigated using principal component analysis, and the suitability of the RUV-III normalization method was determined using coefficients of variation (8, 9). Differential analysis was carried out using Limma packages (10). Sparse partial least squares-discriminant analysis (sPLS-DA) within the mixOmics package was used to identify multivariate minimal protein signatures (11). The Kaplan–Meier survival analysis and Cox proportional hazards models were constructed within R studio (12) using Survival package (13) and plots generated by ggplot2 (14). Data shown are not adjusted for multiple testing. The false discovery rate (FDR) adjusted results were not significant within this cohort.

## Results

### OPSCC Patient Cohort

To investigate protein expression in primary tumors and nodal metastases, we evaluated tissue from a tissue microarray (TMA). The TMA included unmatched specimens from forty-three primary tumors and eleven nodal metastases, and seven matched primary and nodal metastases. All primary tumors were resected from oropharyngeal regions and had squamous cell carcinoma histology (**Table 1**). The TNM staging (8th edition) ranged from T2-4, N0-2, and M0 (**Table 1**). Only one patient within the cohort was HPV-16 positive.

**Table 1:** OPSCC cohort characteristics.

	Nodal Metastasis, n = 11	Primary, n = 43	Matched Nodal Met/Primary, n = 7
**Gender**			
Female	0 (0%)	7 (16%)	1 (14%)
Male	11 (100%)	36 (84%)	6 (86%)
**Age**			
25–50	5 (45%)	5 (12%)	3 (42%)
50–95	6 (55%)	38 (88%)	4 (58%)
**Status**			
Alive	9 (82%)	28 (65%)	2 (29%)
Deceased	2 (18%)	15 (35%)	5 (71%)
**Radiation**			
Yes	3 (27%)	21 (49%)	1 (14%)
No	0	22 (51%)	6 (86%)
N/A	8 (73%)	N/A	N/A
**Adjuvant chemotherapy**			
Yes	1 (9.1%)	8 (19%)	3 (43%)
No	2 (18%)	35 (81%)	4 (57%)
N/A	8 (73%)	N/A	N/A

### Identification of Differentially Expressed Proteins by the Nanostring GeoMX DSP Assay

**Figure 1:**
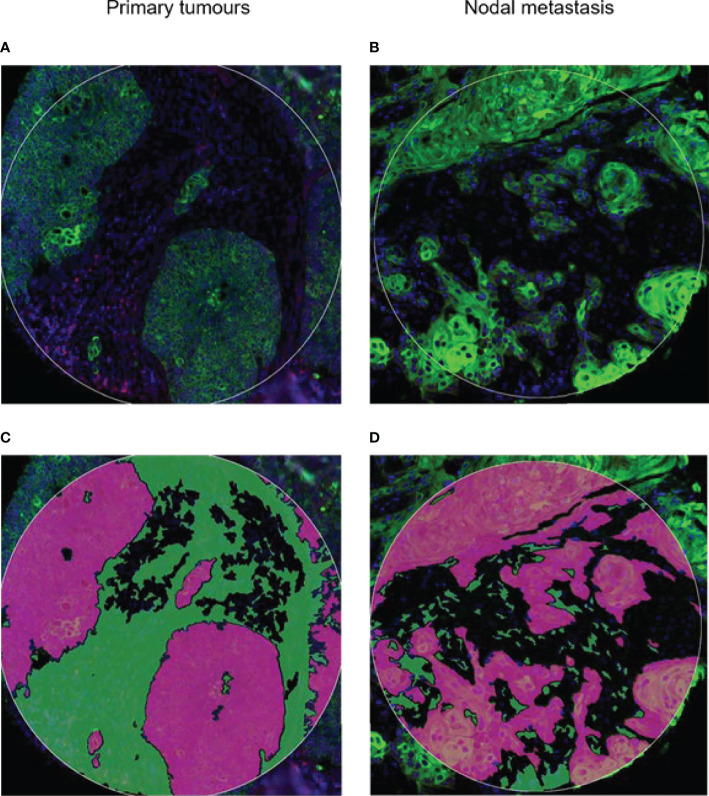
Spatial profiling was performed on tumor microarray cores from **(A)** primary and **(B)** nodal metastasis. Tissues were stained for PanCK^+^ (Tumor) and PanCK^−^ (Stroma) areas. Green, PanCK; Red, CD45. Tissue segmentation strategy to capture **(C)** Tumor mask in purple and **(D)** stromal regions in green. Masks were generated per PanCK^+^/^−^ feature to liberate barcodes for digital counting by nCounter.

**Figure 2:**
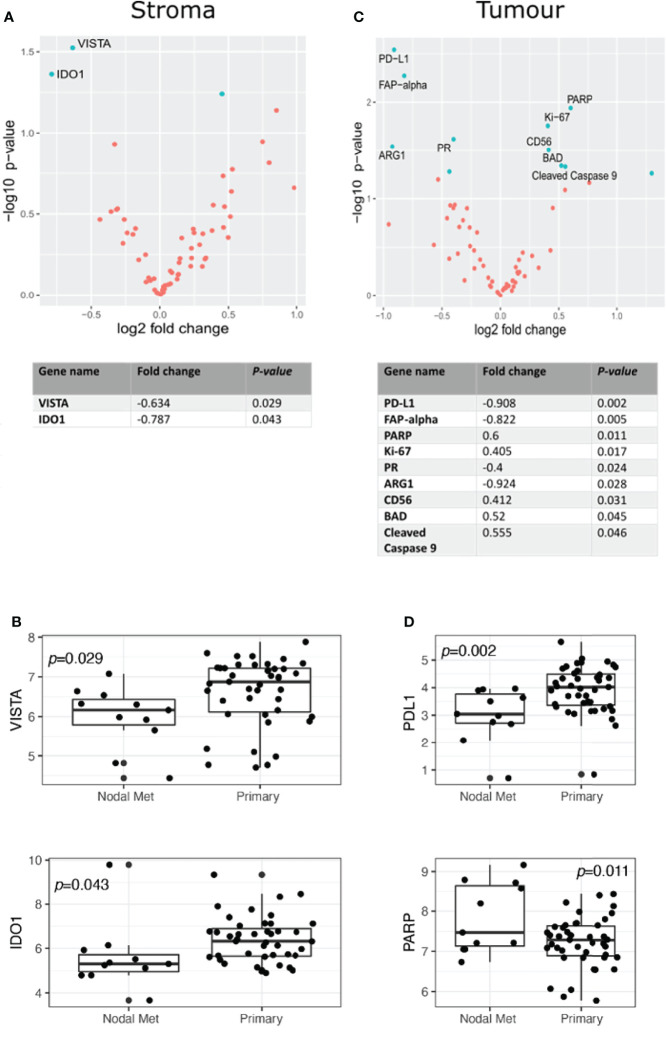
Differential protein expression compared to specimens from unmatched OPSCC primary (n = 43) and nodal metastasis (n = 11). **(A)** Upper panel. Volcano scatter plot showing stromal enrichment of proteins in primary (left) vs nodal metastases (right) ranked by significance (−log10 *P*-value). Lower panel. List of top two significant deregulated proteins ranked by *P*-value. **(B)** Boxplots indicating VISTA and IDO1 enrichment in primary tumors. **(C)** Upper panel. Volcano scatter plot showing tumor region enrichment of proteins from primary (left) vs nodal metastases (right) ranked by significance (−log10 *P*-value). Lower panel. List of top nine significant deregulated proteins ranked by *P*-value. **(D)** Boxplots indicating enrichment of PD-L1 and PARP in primary tumors and nodal metastases, respectively.

**Figure 3:**
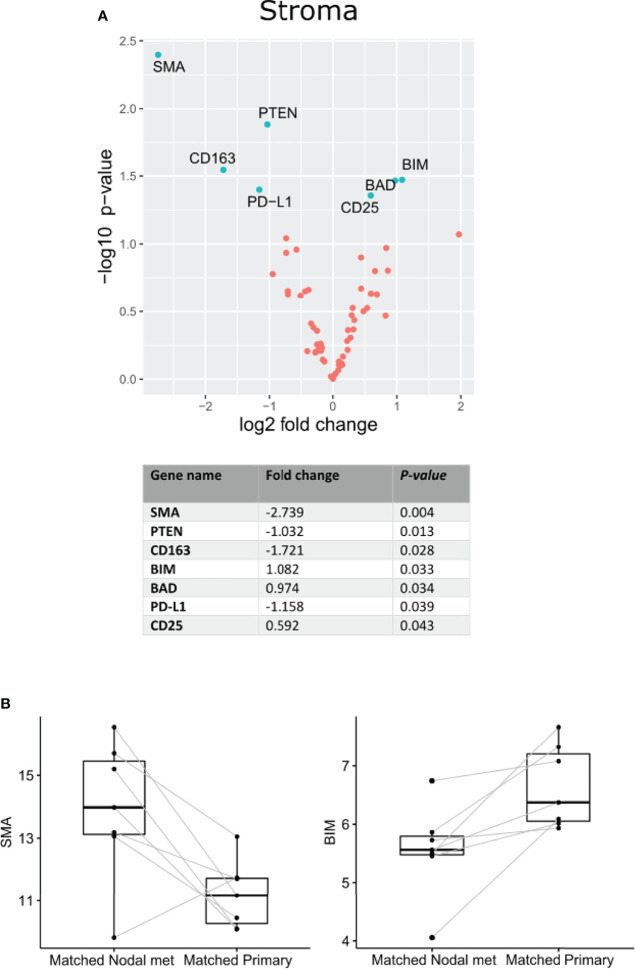
Differential protein expression comparing specimens from matched OPSCC primary tumors and nodal metastasis (n = 7). **(A)** Upper panel. Volcano scatter plot showing stromal enrichment of proteins from nodal metastases (left) vs primary tumors (right) ranked by significance (−log10 *P*-value). Lower panel. List of top seven significant deregulated proteins ranked by *P*-value. **(B)** Representative boxplots indicating SMA and BIM enrichment in matched nodal metastasis and matched primary specimens, respectively.

Nanostring Digital Spatial Profiler (DSP) was applied to investigate the protein expression of 68 TMA cores. By masking on PanCK^+^/PanCK^−^ regions, we compartmentalized protein expression within the tumor and stromal compartments (**Figures 1A–D**). Differential expression (DE) was performed on normalized protein counts to identify compartmental enrichment of proteins in unmatched primary tumors compared to nodal metastases and between patient-matched primary tumors and nodal metastases. We discovered significant differences in key deregulated proteins between the two comparison groups. Additionally, DE within the stroma between unmatched primary tumors and nodal metastasis revealed that V-domain IG suppressor of T cell activation (VISTA) and Indoleamine 2,3-dioxygenase 1 (IDO1) exhibited higher expression in primary tumors (**Figures 2A, B**). Parallel analysis of tumor compartments uncovered DE proteins. Programmed death-ligand 1 (PD-L1), Fibroblast activation protein-alpha (FAP-a), Poly (ADP-ribrose) polymerase (PARP), Ki-67, Progesterone receptor (PR), Arginase 1 (ARG1), CD56, BCL2-antagonist of cell death (BAD), and Cleaved Caspase 9. Accordingly, PD-L1, FAP-a, PR, and ARG1 had higher expression in primary tumors, while PARP, Ki-67, CD56, BAD, and Cleaved Caspase 9 were enriched in nodal metastases (**Figures 2C, D**). Furthermore, analysis of stromal compartments across matched primary and nodal metastasis specimens unveiled seven significant differentially expressed proteins, namely, smooth muscle actin (SMA), phosphatase and tensin homolog deleted on chromosome 10 (PTEN), CD163, Bcl-2-like protein 11 (BIM), BAD, PD-L1, and CD25. BIM, BAD, and CD25 were enriched in matched primary tumors, while SMA, PTEN, CD163, and PD-L1 were higher in matched nodal metastases (**Figures 3A, B**). Analysis of tumor compartments between matched primary and nodal metastasis specimens indicated no significant DE proteins.

### Survival Associations of OPSCC Primary Tumor and Nodal Metastases

**Figure 4:**
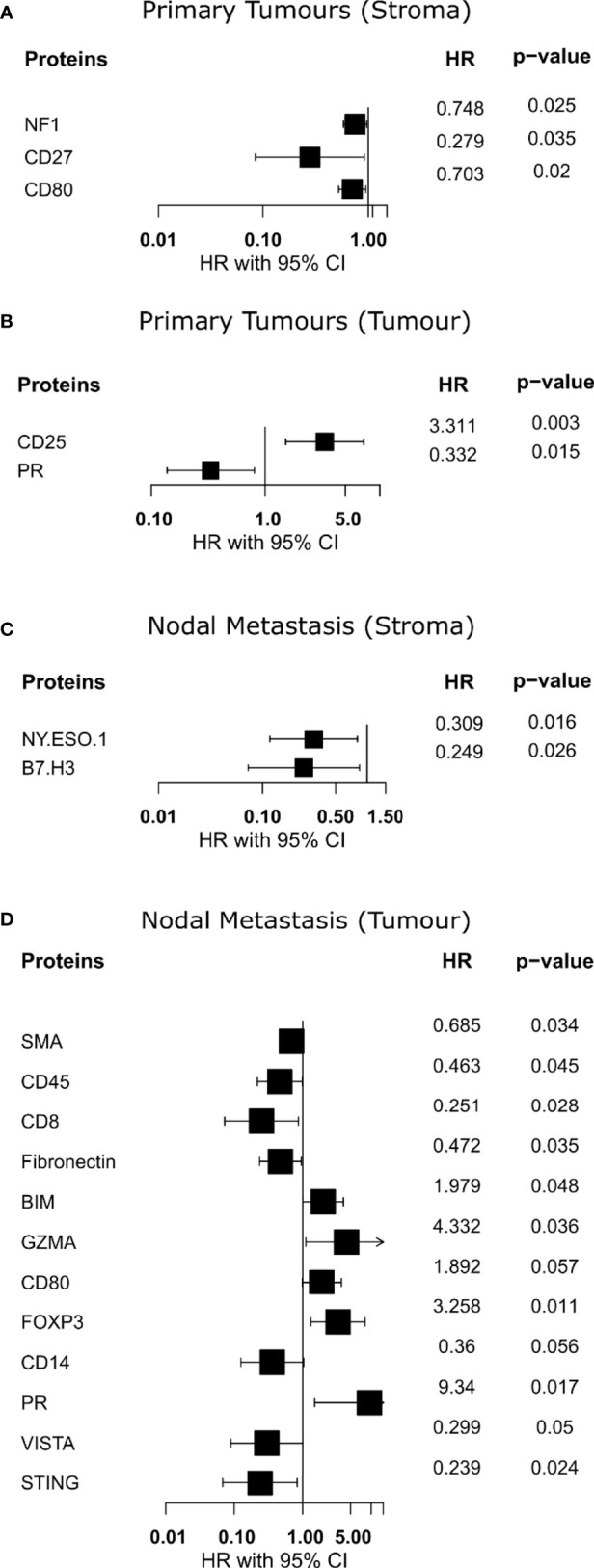
Identification of proteins with overall survival associations. **(A, B)** Forest plot indicating hazard ratio with 95% confidence interval for proteins from primary specimens. **(C, D)** Forest plot indicating hazard ratio with 95% confidence interval for proteins from nodal metastases. HR >1 demonstrates association with poorer outcome.

To assess the association between our protein expression and overall survival (OS), we performed a Cox proportional hazards model on all proteins. Analysis of the stromal compartment in primary tumor specimens revealed that the expression of NF1 (HR = 0.748, p = 0.025), CD27 (HR = 0.279, p = 0.035), and CD80 (HR = 0.703, p = 0.02) was associated with a better OS (**Figure 4A**). Moreover, tumoral compartment analysis across primary tumor samples indicated that PR (HR = 0.332, p = 0.015) was associated with better OS, while CD25 (HR = 3.311, p = 0.003) was associated with worse OS (**Figure 4B**). Survival analysis within the stromal compartment from nodal metastasis specimens showed that NY.ESO.1 (HR = 0.309, p = 0.016), and B7.H3 (HR = 0.249, p = 0.026) were associated with better OS (**Figure 4C**). Evaluation of tumoral compartment of nodal metastasis samples found several proteins associated with better OS, namely, SMA (HR = 0.685, p = 0.034), CD45 (HR = 0.463, p = 0.045), CD8 (HR = 0.251, p = 0.028), Fibronectin (HR = 0.472, p = 0.035), and STING (HR = 0.239, p = 0.024), however, BIM (HR = 1.979, p = 0.048), GZMA (HR = 4.332, p = 0.036), FOXP3 (HR = 3.258, p = 0.011), and PR (HR = 9.34, p = 0.017) were associated with worse OS (**Figure 4D**).

### Multivariate Discrimination of OPSCC Primary Tumors From Nodal Metastases

**Figure 5:**
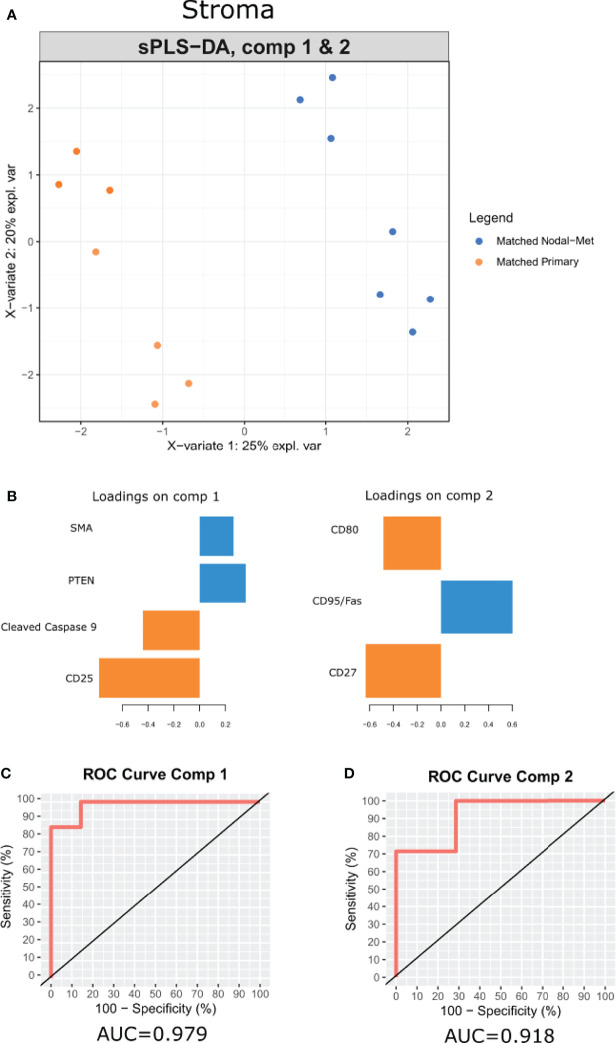
A multi-protein signature differentiates OPSCC tumor progression. **(A)** sPLSDA distinguishes the groups (Matched Primary vs Matched Nodal Mets) by protein signatures in stroma. **(B)** Features of discriminating proteins per component in stroma. **(C, D)** ROC curve of each signature was used to differentiate the groups (Matched Primary vs Matched Nodal Mets). Color of component loadings indicates patient group in which feature was maximally expressed. Positive or negative values in bar chart indicate positive or negative loading to the discriminant signature.

Multivariate analysis by sparse partial least-squares discriminant analysis (sPLSDA) was employed to identify minimal protein signatures that collectively distinguish primary tumors from nodal metastases. Signatures within the stroma of matched primary vs nodal metastases stratified samples effectively (**Figure 5A**). The first signature (**Figure 5B**) included levels of SMA, PTEN, cleaved caspase 9, and CD25 (AUC = 0.979) (**Figure 5C**). A second signature comprised of CD95, CD80, and CD27 distinguished matched sample types as well (AUC = 0.918) (**Figure 5D**).

## Discussion

HNSCC has a high risk of locoregional nodal metastasis, which affects patient prognosis and treatment outcomes (15). Patients with nodal metastasis are considered to have locoregionally advanced disease with a lower chance of remission (16). Currently, the predictors of nodal metastasis include tumor thickness and size, which have been shown to be unreliable predictors (17). It has been difficult to manage clinically negative neck nodes (N0) due to a lack of reliable predictors of occult metastasis (16, 18). In a study conducted by Shah et al., the authors found that there was a 40% chance of nodal metastasis in clinically node-negative neck dissections (19). For the purpose of distinguishing patients with a high risk of nodal metastasis, various pathological and clinical factors, namely, lymphovascular invasion, tumor differentiation, depth of invasion (DOI), and pattern of invasion (POI), have been reported (20, 21). Moreover, modern imaging modalities such as MRI, CT imaging, and PET/CT scanning, have been used to aid in the detection of locoregional nodal metastases. However, some radiographic features, namely, “subclinical”, or “microscopic”, or “occult” disease, remain difficult to diagnose using any of these approaches (22, 23). Therefore, companion diagnostics tools are needed to improve the prediction of the likelihood of the development of locoregional metastasis. Ideally, this would be possible by interrogating the primary tissue to determine its aggressiveness and propensity for metastasis.

Tumor tissue analysis by bulk expression or single cell RNA sequencing offers an overview of the molecular features of HNSCC tumors and their TME. These methods are incapable of revealing the spatial cellular properties required for the anti-tumor immune responses (24). Spatial proteomic approaches can provide compartment-specific tumor information to aid in delineating tumor composition. To garner insight into these properties that distinguish primary OPSCC tumors from their metastatic nodal counterparts, we have employed Digital Spatial Profiling to address a targeted profile of proteins and present this data as a first step in profiling HNSCC nodal involvement in an OPSCC cohort.

Unmatched analyses between primary tumors (n = 43) and nodal metastases (n = 11) provided insight into the potential dysregulation of several proteins, despite the inherent limitations associated with such a sampling strategy. VISTA, IDO1, and PD-L1 are key immune checkpoints, with VISTA and IDO1 appearing more abundant in the stromal compartment of primary tumors, whereas PD-L1 indicated higher expression in their respective tumor compartments. The V-domain Ig suppressor of T cell activation (VISTA) is an inhibitory immune checkpoint protein that is typically expressed on naïve CD4^+^ and Foxp3^+^ Tregs and functions by inhibiting T-cell proliferation and promoting naive to Treg conversion (25). VISTA was associated with several immune cell regions in the stroma but not in HNSCC tumors (26). Blockade of VISTA was found to boost anti-tumor immunity in the tumor microenvironment by increasing the number of activated dendritic cells (DCs) and decreasing the number of myeloid-derived suppressor cells (MDSCs). IDO1 induces T-cell apoptosis through activation of caspase 8 and releases mitochondrial cytochrome C, functioning in an immunosuppressive capacity (27). Programmed death-ligand 1 (PD-L1) is a canonical inhibitory immune checkpoint that binds PD-1 on the surface of tumors and immune cells (28). PD-L1 expression on the surface of HNSCC tumor cells is associated with a more robust anti-tumor immune response (29, 30).

Apoptotic pathways play an important role in tumorigenesis, and our results indicate that pro-apoptotic BAD and cleaved caspase 9 were enriched in tumor cells of unmatched nodal metastases. Conversely, in the matched analysis, BIM and BAD were enriched in the stroma of primary tumors. Under various physiological and patho-physiological conditions, Bcl-2 interacting mediator of cell death (BIM) promotes the intrinsic apoptotic pathway (31). Bcl2-associated agonist of cell death (BAD) is a member of the BCL2 family of proteins that act as pro-apoptotic regulators (32). The expression of BAD has been linked to chemoresistance in cancer patients (33, 34). Caspase 9 functions as a pro-apoptotic regulator, allowing the activation of effector caspases 3 and 7 (35). Caspase 9-induced apoptosis has been linked to chemotherapy response. Studies have shown that HNSCC tumors may be resistant to cisplatin if they have a reduced expression of caspase 9 (36).

It is interesting to note that despite an imbalance in samples in the unmatched analysis, several proteins appear enriched within nodal metastases relative to primary tumors. In addition to the pro-apoptotic markers above, Ki-67 and PARP appear enriched in nodal tumor cells. Ki-67 is an established proliferation marker (37), while PARP responds to DNA damage by recruiting effector proteins to repair single-strand breaks (38, 39). PARP inhibitors have been studied as a promising drug to overcome the limitations of conventional therapies that cause DNA damage, such as chemotherapy or radiotherapy (40). This pro-apoptotic, proliferative, and DNA damage phenotype of unmatched nodal metastatic cells is a novel finding in our data that requires further validation, perhaps indicating tumor evolution or response to changes in the cellular ecosystem of the lymph node.

Several other notable features of our data include increased expression of CD25 in the stroma of matched primary tumors. CD25, also known as the IL-2 receptor alpha, is a protein found on activated T cells, specifically Tregs (41). Interestingly, we found that it was CD25 expression within primary tumor regions, not stroma, that was associated with poorer OS. Additionally, PR expression appeared associated with better survival within primary tumors and was also enriched within their tumor regions relative to nodal metastases.

In addition to the differential expression of each individual protein, we applied a multivariate statistical model (sPLSDA) to further discern features that collectively discriminated between our matched patient samples. Of note, this model only performed effectively in stratifying these sample types by their stroma. Expression of CD25 and cleaved caspase 9 in the primary samples and PTEN and SMA in nodal samples could discern samples. Similarly, levels of CD80 and CD27 within primary samples and CD95 in nodal samples could separate these samples. This method offers an alternative to traditional differential expression that may provide insight into contributing differences in observed phenotypes using a multivariate approach.

A protein association with overall survival (OS) was investigated using the Univariate Cox proportional hazards model. Stromal expression of CD27 in primary tumor specimens, and NY-ESO.1 and B7-H3 expression in the nodal metastasis samples, was associated with improved OS. In nodal metastases tumor regions, CD8 and STING were associated with improved OS; however, GZMA was associated with poorer OS. Interestingly, the expression of PR in the tumoral compartment of primary and nodal metastasis tumors demonstrated a different survival pattern. Although the PR expression was associated with improved OS in primary tumors, it was associated with worse OS in nodal metastasis specimens. Immune response protein markers, CD27, and NY.ESO.1 within the stromal compartment of primary and nodal metastasis specimens, were associated with improved OS in our study. Cluster of differentiation 27 (CD27) belongs to the tumor necrosis factor (TNF) receptor superfamily and is involved in T and B cell co-stimulation (42). New York esophageal squamous cell carcinoma 1 (NY.ESO.1) is a member of the cancer testis antigen (CTA) family, which regulates both humoral and cellular immune responses. NY-ESO.1 expression has been linked to higher tumor differentiation grade and stage, and lymph node metastasis (43). In our study, we found that B7.H3 and STING protein expression, NF-κB pathway markers, were linked to improved OS. B7.H3, also known as CD276, promotes anti-tumor immune response by activating T and NK cells (44). STING, on the other hand, contributes to the immune response to tumor cells through the upregulation of interferon gamma 1 (IFN1) (45). Progesterone receptor (PR) is a type of androgen receptor and a member of the nuclear receptor family of transcription factors that regulates target gene expression networks in response to its ligand progesterone (46).

Our study has identified tumor and stromal compartment-specific proteins and signatures that may have predictive and prognostic implications for HNSCC and the development of nodal metastasis. Nevertheless, the study is impacted by the number of samples for each cohort, in particular the matched group. We propose further investigation to profile primary, locoregional, and distant metastasis from matched patient samples to understand the molecular features driving the development of metastasis in OPSCC.

## Data Availability Statement

The data presented in the study are deposited in the Geo repository, under accession number GEO Submission GSE200601.

## Ethics Statement

This study has the approval from the Queensland University of Technology Human Research Ethics Committee (UHREC #2000000494). The patients/participants provided their written informed consent to participate in this study.

## Author Contributions

Experimental design: HR, BH, and AK. Methodology: HR, JM, AM, BH, and AK. Data analysis and reporting: HR, JM, AM, KO, BGMH, and AK. Manuscript preparation and critical review: all authors. All authors listed have made a substantial, direct, and intellectual contribution to the work and approved it for publication.

## Funding

This project is funded by the Garnett Passe and Rodney Williams Memorial Foundation (GPRWMF) conjoint grant for AK and BH. AK is supported by a fellowship from the NHMRC (1157741). KO’B and AK are supported by the PA Research Foundation.

## Conflict of Interest

AM is employed by QCIF Bioinformatics.

The remaining authors declare that the research was conducted in the absence of any commercial or financial relationships that could be construed as a potential conflict of interest.

## Publisher’s Note

All claims expressed in this article are solely those of the authors and do not necessarily represent those of their affiliated organizations, or those of the publisher, the editors and the reviewers. Any product that may be evaluated in this article, or claim that may be made by its manufacturer, is not guaranteed or endorsed by the publisher.

## References

[1] Johnson DE Burtness B Leemans CR Lui VWY Bauman JE Grandis JR. Head and Neck Squamous Cell Carcinoma. Nat Rev Dis Primers (2020) 6(1):92. doi: 10.1038/s41572-020-00224-3 33243986 PMC7944998

[2] Sabatini ME Chiocca S. Human Papillomavirus as a Driver of Head and Neck Cancers. Br J Cancer (2020) 122(3):306–14. doi: 10.1038/s41416-019-0602-7 PMC7000688 31708575

[3] Chen T-C Wu C-T Ko J-Y Yang T-L Lou P-J Wang C-P. Clinical Characteristics and Treatment Outcome of Oropharyngeal Squamous Cell Carcinoma in an Endemic Betel Quid Region. Sci Rep (2020) 10(1):526. doi: 10.1038/s41598-019-57177-1 31949181 PMC6965138

[4] Argiris A Karamouzis MV Raben D Ferris RL. Head and Neck Cancer. Lancet (2008) 371(9625):1695–709. doi: 10.1016/S0140-6736(08)60728-X PMC7720415 18486742

[5] Sadeghi Rad H Monkman J Warkiani ME Ladwa R O'Byrne K Rezaei N. Understanding the Tumor Microenvironment for Effective Immunotherapy. Medicinal Res Rev (2021) 41(3):1474–98. doi: 10.1002/med.21765 PMC8247330 33277742

[6] Stern PL Dalianis T. Oropharyngeal Squamous Cell Carcinoma Treatment in the Era of Immune Checkpoint Inhibitors. Viruses (2021) 13(7):1234. doi: 10.3390/v13071234 34202255 PMC8310271

[7] Kulasinghe A Taheri T O’Byrne K Hughes BGM Kenny L Punyadeera C. Highly Multiplexed Digital Spatial Profiling of the Tumor Microenvironment of Head and Neck Squamous Cell Carcinoma Patients. Front Oncol (2021) 10. doi: 10.3389/fonc.2020.607349 PMC7851078 33542903

[8] Risso D Ngai J Speed TP Dudoit S. Normalization of RNA-Seq Data Using Factor Analysis of Control Genes or Samples. Nat Biotechnol (2014) 32(9):896–902. doi: 10.1038/nbt.2931 25150836 PMC4404308

[9] Molania R Gagnon-Bartsch JA Dobrovic A Speed TP. A New Normalization for Nanostring Ncounter Gene Expression Data. Nucleic Acids Res (2019) 47(12):6073–83. doi: 10.1093/nar/gkz433 PMC6614807 31114909

[10] Ritchie ME Phipson B Wu D Hu Y Law CW Shi W. Limma Powers Differential Expression Analyses for RNA-Sequencing and Microarray Studies. Nucleic Acids Res (2015) 43(7):e47. doi: 10.1093/nar/gkv007 25605792 PMC4402510

[11] Rohart F Gautier B Singh A KA LC. Mixomics: An R Package for 'Omics Feature Selection and Multiple Data Integration. PloS Comput Biol (2017) 13(11):e1005752. doi: 10.1371/journal.pcbi.1005752 29099853 PMC5687754

[12] Team R. RStudio: Integrated Development for R. Boston, MA: RStudio (2020). Available at: http://www.rstudio.com/.

[13] Therneau TM. A Package for Survival Analysis in R (2021). Available at: https://CRAN.R-project.org/package=survival.

[14] Hadley W. Ggplot2: Elegrant Graphics for Data Analysis. Springer (2016).

[15] Chow LQM. Head and Neck Cancer. N Engl J Med (2020) 382(1):60–72. doi: 10.1056/NEJMra1715715 31893516

[16] Ampil FL Nathan CA Sangster G Caldito G. Head and Neck Cancer With Lower Neck Nodal Metastases: Management of 23 Cases and Review of the Literature. Oral Oncol (2012) 48(4):325–8. doi: 10.1016/j.oraloncology.2011.11.016 22405883

[17] Puram SV Tirosh I Parikh AS Patel AP Yizhak K Gillespie S. Single-Cell Transcriptomic Analysis of Primary and Metastatic Tumor Ecosystems in Head and Neck Cancer. Cell (2017) 171(7):1611–24.e24. doi: 10.1016/j.cell.2017.10.044 29198524 PMC5878932

[18] De Silva RK Siriwardena B Samaranayaka A Abeyasinghe W. Tilakaratne WM. A Model to Predict Nodal Metastasis in Patients With Oral Squamous Cell Carcinoma. PloS One (2018) 13(8):e0201755. doi: 10.1371/journal.pone.0201755 30091996 PMC6084951

[19] Shah JP. Patterns of Cervical Lymph Node Metastasis From Squamous Carcinomas of the Upper Aerodigestive Tract. Am J Surg (1990) 160(4):405–9. doi: 10.1016/S0002-9610(05)80554-9 2221244

[20] Tai SK Li WY Yang MH Chu PY Wang YF. Perineural Invasion in T1 Oral Squamous Cell Carcinoma Indicates the Need for Aggressive Elective Neck Dissection. Am J Surg Pathol (2013) 37(8):1164–72. doi: 10.1097/PAS.0b013e318285f684 23681077

[21] Rajapakshe RM Pallegama RW Jayasooriya PR Siriwardena BS Attygalla AM Hewapathirana S. A Retrospective Analysis to Determine Factors Contributing to the Survival of Patients With Oral Squamous Cell Carcinoma. Cancer Epidemiol (2015) 39(3):360–6. doi: 10.1016/j.canep.2015.02.011 25779678

[22] Sohn B Koh YW Kang WJ Lee JH Shin NY Kim J. Is There an Additive Value of 18 F-FDG PET-CT to CT/MRI for Detecting Nodal Metastasis in Oropharyngeal Squamous Cell Carcinoma Patients With Palpably Negative Neck? Acta Radiol (2016) 57(11):1352–9. doi: 10.1177/0284185115587544 26013025

[23] Kann BH Aneja S Loganadane GV Kelly JR Smith SM Decker RH. Pretreatment Identification of Head and Neck Cancer Nodal Metastasis and Extranodal Extension Using Deep Learning Neural Networks. Sci Rep (2018) 8(1):14036. doi: 10.1038/s41598-018-32441-y 30232350 PMC6145900

[24] Sadeghi Rad H Bazaz SR Monkman J Ebrahimi Warkiani M Rezaei N O'Byrne K. The Evolving Landscape of Predictive Biomarkers in Immuno-Oncology With a Focus on Spatial Technologies. Clin Trans Immunol (2020) 9(11):e1215. doi: 10.1002/cti2.1215 PMC7680923 33251010

[25] ElTanbouly MA Croteau W Noelle RJ Lines JL. VISTA: A Novel Immunotherapy Target for Normalizing Innate and Adaptive Immunity. Semin Immunol (2019) 42:101308. doi: 10.1016/j.smim.2019.101308 31604531 PMC7233310

[26] Lines JL Pantazi E Mak J Sempere LF Wang L O'Connell S. VISTA is an Immune Checkpoint Molecule for Human T Cells. Cancer Res (2014) 74(7):1924–32. doi: 10.1158/0008-5472.CAN-13-1504 PMC3979527 24691993

[27] Fallarino F Grohmann U Vacca C Bianchi R Orabona C Spreca A. T Cell Apoptosis by Tryptophan Catabolism. Cell Death Differ (2002) 9(10):1069–77. doi: 10.1038/sj.cdd.4401073 12232795

[28] Liebl MC Hofmann TG. Identification of Responders to Immune Checkpoint Therapy: Which Biomarkers Have the Highest Value? J Eur Acad Dermatol Venereol (2019) 33:52–6. doi: 10.1111/jdv.15992 31833606

[29] Cohen EE Soulières D Le Tourneau C Dinis J Licitra L Ahn M-J. Pembrolizumab Versus Methotrexate, Docetaxel, or Cetuximab for Recurrent or Metastatic Head-and-Neck Squamous Cell Carcinoma (KEYNOTE-040): A Randomised, Open-Label, Phase 3 Study. Lancet (2019) 393(10167):156–67. doi: 10.1016/S0140-6736(18)31999-8 30509740

[30] Brahmer JR Tykodi SS Chow LQ Hwu W-J Topalian SL Hwu P. Safety and Activity of Anti–PD-L1 Antibody in Patients With Advanced Cancer. N Engl J Med (2012) 366(26):2455–65. doi: 10.1056/NEJMoa1200694 PMC3563263 22658128

[31] Shukla S Saxena S Singh BK Kakkar P. BH3-Only Protein BIM: An Emerging Target in Chemotherapy. Eur J Cell Biol (2017) 96(8):728–38. doi: 10.1016/j.ejcb.2017.09.002 29100606

[32] Boac BM Abbasi F Ismail-Khan R Xiong Y Siddique A Park H. Expression of the BAD Pathway is a Marker of Triple-Negative Status and Poor Outcome. Sci Rep (2019) 9(1):1–14. doi: 10.1038/s41598-019-53695-0 31767884 PMC6877530

[33] Bansal N Marchion DC Bicaku E Xiong Y Chen N Stickles XB. BCL2 Antagonist of Cell Death Kinases, Phosphatases, and Ovarian Cancer Sensitivity to Cisplatin. J Gynecol Oncol (2012) 23(1):35–42. doi: 10.3802/jgo.2012.23.1.35 22355465 PMC3280065

[34] Marchion DC Cottrill HM Xiong Y Chen N Bicaku E Fulp WJ. BAD Phosphorylation Determines Ovarian Cancer Chemosensitivity and Patient Survival. Clin Cancer Res (2011) 17(19):6356–66. doi: 10.1158/1078-0432.CCR-11-0735 PMC3186862 21849418

[35] Thornberry NA Lazebnik Y. Caspases: Enemies Within. Science (1998) 281(5381):1312–6. doi: 10.1126/science.281.5381.1312 9721091

[36] Kuwahara D Tsutsumi K Oyake D Ohta T Nishikawa H Koizuka I. Inhibition of Caspase-9 Activity and Apaf-1 Expression in Cisplatin-Resistant Head and Neck Squamous Cell Carcinoma Cells. Auris Nasus Larynx (2003) 30:85–8. doi: 10.1016/S0385-8146(02)00129-3 12543167

[37] Sun X Kaufman PD. Ki-67: More Than a Proliferation Marker. Chromosoma (2018) 127(2):175–86. doi: 10.1007/s00412-018-0659-8 PMC5945335 29322240

[38] Langelier M-F Riccio AA Pascal JM. PARP-2 and PARP-3 are Selectively Activated by 5′ Phosphorylated DNA Breaks Through an Allosteric Regulatory Mechanism Shared With PARP-1. Nucleic Acids Res (2014) 42(12):7762–75. doi: 10.1093/nar/gku474 PMC4081085 24928857

[39] Eustermann S Wu W-F Langelier M-F Yang J-C Easton LE Riccio AA. Structural Basis of Detection and Signaling of DNA Single-Strand Breaks by Human PARP-1. Mol Cell (2015) 60(5):742–54. doi: 10.1016/j.molcel.2015.10.032 PMC4678113 26626479

[40] Lord CJ Ashworth A. PARP Inhibitors: Synthetic Lethality in the Clinic. Science (2017) 355(6330):1152–8. doi: 10.1126/science.aam7344 PMC6175050 28302823

[41] Flynn MJ Hartley JA. The Emerging Role of Anti-CD 25 Directed Therapies as Both Immune Modulators and Targeted Agents in Cancer. Br J Haematol (2017) 179(1):20–35. doi: 10.1111/bjh.14770 28556984

[42] Starzer AM Berghoff AS. New Emerging Targets in Cancer Immunotherapy: CD27 (Tnfrsf7). ESMO Open (2019) 4:e000629. doi: 10.1136/esmoopen-2019-000629 PMC7082637 32152062

[43] Thomas R Al-Khadairi G Roelands J Hendrickx W Dermime S Bedognetti D. NY-ESO-1 Based Immunotherapy of Cancer: Current Perspectives. Front Immunol (2018) 9:947. doi: 10.3389/fimmu.2018.00947 29770138 PMC5941317

[44] Luo L Chapoval AI Flies DB Zhu G Hirano F Wang S. B7-H3 Enhances Tumor Immunity *In Vivo* by Costimulating Rapid Clonal Expansion of Antigen-Specific CD8+ Cytolytic T Cells. J Immunol (2004) 173(9):5445–50. doi: 10.4049/jimmunol.173.9.5445 15494491

[45] Ishikawa H Barber GN. STING Is an Endoplasmic Reticulum Adaptor That Facilitates Innate Immune Signalling. Nature (2008) 455(7213):674–8. doi: 10.1038/nature07317 PMC2804933 18724357

[46] Grimm SL Hartig SM Edwards DP. Progesterone Receptor Signaling Mechanisms. J Mol Biol (2016) 428(19):3831–49. doi: 10.1016/j.jmb.2016.06.020 27380738

